# Changes in Health-Related Quality of Life After Transient Ischemic Attack

**DOI:** 10.1001/jamanetworkopen.2021.17403

**Published:** 2021-07-20

**Authors:** Irene L. Katzan, Andrew Schuster, Lynn Daboul, Christine Doherty, Sidra Speaker, Ken Uchino, Brittany Lapin

**Affiliations:** 1 Neurological Institute, Cleveland Clinic, Cleveland, Ohio; 2 Cleveland Clinic Lerner College of Medicine, Cleveland, Ohio

## Abstract

**Importance:**

Numerous studies have found that patients diagnosed with TIA have decreased health-related quality of life, which has been interpreted as suggesting that patients with TIA have residual symptoms after the event. Studies assessing health status in the same patients before and after an event are lacking but may allow a direct determination of the association of TIA with postevent health status.

**Objective:**

To examine patient-reported health before transient ischemic attack (TIA) among individuals diagnosed with this event and evaluate change in patient-reported health after the event overall and by TIA characterization subgroups.

**Design, Setting, and Participants:**

This cohort study was conducted among 236 patients with a clinical diagnosis of TIA from October 2015 to December 2017 in a large US health system that collects a patient-reported outcome measure in ambulatory setting as part of routine care. Included patients had patient-reported global health scale assessments completed as part of routine care before and after a TIA event. Data were analyzed from March through July 2020.

**Main Outcomes and Measures:**

The main outcome was Patient-Reported Outcome Measurement Information System Global Health (PROMIS GH) scale score before and after TIA. A change of 5 or more points in this score is considered clinically relevant. The secondary outcomes included change in patient-reported global health by clinical impression of the probability of a TIA event, pattern of neurological deficits, and short-term risk of stroke, as assessed by the ABCD2 score.

**Results:**

Among 263 patients who experienced TIA, mean (SD) age was 67.9 (13.4) years and 138 (52.5%) were women. The median (interquartile range) time between patient-reported global health scores was 152 (94-284) days. Mean (SD) baseline patient-reported global physical health and mental health scale summary scores were 43.4 (8.2) and 47.7 (9.7), respectively, and were statistically significantly decreased compared with the general population mean (SD) scores of 50 (10; *P* < .001) for physical and mental health. The difference between physical health summary score among study participants and the general population was clinically relevant. Mean (SD) summary scores were not statistically significantly different after the event compared with before the event overall (physical health: 44.1 [8.2], for a mean [SE] improvement of 0.65 [0.38] points; *P* = .09; mental health: 47.4 [9.1], for a mean [SE] worsening of 0.25 [0.38] points; *P* = .51) or within subgroups.

**Conclusions and Relevance:**

These findings suggest that impaired health status among patients diagnosed with TIA reflect, at least in part, an impaired premorbid state of health. This study did not find that TIA events were associated with worsening of health status overall or within subgroups.

## Key Points

**Question:**

Does health-related quality of life (HRQOL) worsen after transient ischemic attack (TIA)?

**Findings:**

In this cohort study of 263 patients who completed a patient-reported assessment of their global health as part of routine care before and after TIA, mean baseline physical health summary score was statistically significantly decreased compared with the general population, and the difference was clinically relevant. Physical health and mental health summary scores were not statistically significantly different after the event compared with before the event.

**Meaning:**

These findings suggest that the impaired HRQOL found among patients diagnosed with TIA reflects an impaired premorbid state of health rather than worsening health after the TIA event.

## Teaser

This cohort study investigates changes in patient-reported global health as measured before vs after transient ischemic attack.

## Introduction

Symptoms of a transient ischemic attack (TIA), by definition, resolve completely within 24 hours. Numerous studies, however, have found that patients diagnosed with TIA have symptoms, including depression,^1^ fatigue,^2^ cognitive impairment,^3^ and reduced health-related quality of life (HRQOL).^4^ These findings have been interpreted as suggesting that patients with TIA have residual psychological and physical adverse outcomes after the event.^5,6^

Previous studies^5,7^ included patients with minor stroke, obscuring outcomes of patients with TIA, and lacked comparator groups of individuals with similar characteristics, making it difficult to know if the poor health status of patients with TIA was associated with their underlying characteristics rather than the event.^5^ Proposed alternative explanations for poor outcomes among TIA patients include subsequent stroke^8,9^ and treatments administered after the event.^10^ To our knowledge, no studies have assessed health status in the same patients before and after the event, which may allow a direct determination of the association of TIA with postevent patient health. Additional evaluation of the association of TIA with subsequent patient health is clearly warranted.^3,5^

To gain insight on the reasons for the impaired HRQOL seen among patients diagnosed with TIA, we performed a retrospective cohort study of patients who had a patient-reported global health (GH) scale evaluation completed as part of routine care before and after TIA. The primary aims were to determine patient-reported GH scale mental health (MH) and physical health (PH) summary scores prior to a TIA and quantify changes in scores after the event. Our secondary aims were to determine changes in patient-reported GH scale scores after TIA stratified by the clinical impression of the probability of a TIA event, pattern of neurological deficits, and short-term risk of stroke and determine association between worsening PH and MH and the following clinical factors: interval stroke, new or worsening medical conditions, hospital admissions, and medication changes.

## Methods

This cohort study was approved by the Cleveland Clinic Institutional Review Board. Because this study used data obtained as part of standard of care and did not include patient contact, the requirement for informed consent was waived. This study followed the Strengthening the Reporting of Observational Studies in Epidemiology (STROBE) reporting guideline.

Our patient-reported data collection processes have been described previously.^11^ As part of routine outpatient care, patient-reported data are collected within the health system^12^ via tablets at the time of the ambulatory visit or through the electronic health record (EHR) patient portal MyChart (Epic Systems) before the appointment. All areas using electronic patient-reported data collect the patient-reported GH scale score in addition to condition-specific scales.

All patients who had an inpatient or outpatient encounter diagnosis of TIA from October 2015 to December 2017 were identified from an administrative data set and merged with a data set of all patients with patient-reported GH scale scores. A manual EHR review was performed to determine final study eligibility. Included patients were aged 18 years or older with an event diagnosed as TIA from October 2015 to December 2017 who had a patient-reported GH scale score determined before and after the event date. Exclusion criteria included (1) acute infarct on brain magnetic resonance imaging (MRI) or head computed tomography (CT) performed at the time of initial evaluation or evidence of stroke referable to TIA symptoms on imaging done within 1 year after the event, (2) repetitive episodes of symptoms making it difficult to identify the date of a TIA event, (3) persistent neurological symptoms inconsistent with the definition for TIA, and (4) clear alternative diagnoses for the event, including migraine, epilepsy, hyperventilation, cardiac syncope, or hypoglycemia, despite having been given the encounter diagnosis of TIA. Patients with imaging evidence of stroke were excluded based on the currently accepted tissue-based definition of TIA.^13^ We then fully abstracted EHRs of patients meeting the inclusion and exclusion criteria to obtain all study variables.

### Study Variables

#### Patient-Reported Outcome Measure

Evaluations of GH were completed using the Patient-Reported Outcome Measurement Information System Global Health (PROMIS GH; US Department of Health and Human Services) scale, a common patient-reported measure used to assess GH and an outcome measure for patients with stroke recommended by the International Consortium for Health Outcomes Measurement.^14^ It comprises 10 global items from which MH and PH summary scores are computed. Scores are standardized to the general population on a *T* scale.^15^ Mean (SD) *T* score is 50 (10); higher scores indicate better patient-reported health. For MH, the possible *T* score ranges from 21.3 to 67.6. For PH, the possible *T* score ranges from 17.1 to 67.7. A change of 5 or more points is considered conservatively to be a clinically relevant difference.^16,17,18^

#### Clinical Variables

We manually abstracted EHRs of patients in the study cohort for variables that could potentially impact baseline or follow-up patient-reported GH scale scores. The data were stored in the Research Electronic Data Capture online database system hosted at Cleveland Clinic.^19^

##### TIA Characterization

Clinician clinical impression of TIA event as documented in the EHR was categorized as probable TIA vs possible TIA vs not documented. Because patients could be evaluated by clinicians from different specialties, this variable was further categorized as neurological vs nonneurological impression. The pattern of deficits was categorized as focal, nonfocal, and mixed using the definitions of Bos et al.^20^

The ABCD2 (age, blood pressure, clinical symptoms, duration, diabetes) stroke risk score was also retrospectively abstracted. This clinician-reported 5-item scale (scale range, 0-7) classifies risk of subsequent stroke within the first 90 days after the TIA.^21^ Although this score is not specifically designed to determine whether clinical events represent TIA, we considered events with a higher short-term risk of stroke (ie, stroke risk score ≥4) more likely to represent episodes of cerebrovascular ischemia.^22,23^

Imaging variables included performance of brain MRI with diffusion-weighted imaging (DWI) and head CT up to 2 weeks after the clinical event and presence of an acute infarct. Completion of MRI and CT from 2 weeks to 1 year after the event was also recorded; 1 author (I.L.K.) reviewed all images in which stroke was present to determine whether the infarct location was referable to the TIA.

##### Medical Information

Selected medical conditions present prior to baseline patient-reported GH scale score completion, which could have potentially impacted baseline health status scores, were abstracted from encounter notes, problem lists, and the medical history section of the EHR. New or worsening medical conditions occurring between the pre-TIA and post-TIA patient-reported GH scale score that could be associated with post-TIA health status outcomes were also abstracted. Definitions and methods for EHR abstraction were defined in an abstraction manual. Audits of EHRs were performed in 12 patient records (4.6%). Variables with low reliability in these EHR audits (ie, variables with lack of agreement between initial measure and reabstraction among more than 2 patients), including pattern of symptoms and changes in medical conditions, were reabstracted for all patients.

### Statistical Analysis

Characteristics of the study cohort were summarized using descriptive statistics. For patients who had more than 1 patient-reported GH scale score before or after TIA, scores completed closest to the TIA were included in analyses. Patient-reported GH scale summary scores were compared with the population mean (SD) of 50 (10) using 1-sided, 1-sample *t* test. Change in patient-reported GH scale PH and MH scores and individual global items from before to after the event was assessed using paired *t* test. The proportion of patients with deterioration or improvement of 5 or more points in patient-reported GH scale PH or MH summary scores from before to after TIA were calculated overall and stratified by the following categories: clinical impression of TIA (ie, probable vs possible), pattern of neurological deficits (ie, focal, nonfocal, or mixed),^20^ risk of future stroke (ie, high risk: stroke risk score ≥4 vs lower risk: stroke risk score <4).^21^ *T* scores and change scores were compared between groups using *t* test or Mann-Whitney U test, as appropriate.

Univariable logistic regression models were used to evaluate the association of individual factors with worsening in patient-reported GH scale score (ie, clinically relevant deterioration at ≥5 points) overall and by subgroup. We constructed 2 multivariable logistic regression models to evaluate the association of a limited set of prespecified variables with worsening by 5 points or more in the patient-reported GH scale MH and PH scores. Variables in these models included age, sex, race, marital status, pattern of deficit (ie, focal, nonfocal, or mixed), stroke risk score of 4 or greater, and time between patient-reported GH scale scores. Race was obtained from the EHR, and the race variable was used to characterize the population and assess the representativeness of the sample.

We performed 3 sensitivity analyses. First, to better understand potential selection bias of the study cohort, which required completion of patient-reported GH scale evaluation before and after TIA for inclusion, we compared postevent patient-reported GH scale scores among patients seen in the cerebrovascular center who had pre-event patient-reported GH scale scores with postevent GH scores of patients seen in the center who lacked pre-event scores. Patients in these groups were diagnosed with TIA by a vascular neurologist and had the date of TIA recorded in structured fields within the EHR. Second, to determine whether patient-reported GH scale was sensitive to change among patients with transient symptoms who had imaging evidence of stroke, we calculated the proportion of patients who had worsening in patient-reported GH scale scores among 10 patients with transient symptoms who were excluded from the cohort because of acute infarct in imaging. Third, to determine whether inclusion of patients without MRI was associated with confounding by including some patients with stroke rather than TIA,^24^ we repeated calculation of change in patient-reported GH scale scores in the subgroup of patients with MRI results as part of the TIA evaluation.

Significance was established throughout at *P* < .05. Given that the results of our study are hypothesis generating and focused on magnitudes of association, there was no formal adjustment for multiple comparisons. Statistical analyses were conducted using SAS statistical software version 9.4 (SAS Institute Inc). Data were analyzed from March through July 2020.

## Results

Among 1032 patients with any encounter diagnosis of TIA recorded in the EHR from October 12, 2015, to December 25, 2017, who had 2 or more patient-reported GH scale scores during that time period, 263 patients had an event meeting inclusion criteria and were included in the final cohort (Figure). The mean (SD) age of the study cohort was 67.9 (13.4) years, and 138 (52.5%) were women; most patients were White (223 patients [87.1%]) (Table 1). Among 158 patients (60.1%), DWI MRI was done as part of the evaluation (eTable 1 in the Supplement). Median (interquartile range; range) time between patient-reported GH scale scores was 152 (94-284; 16-780) days.

**Figure.**
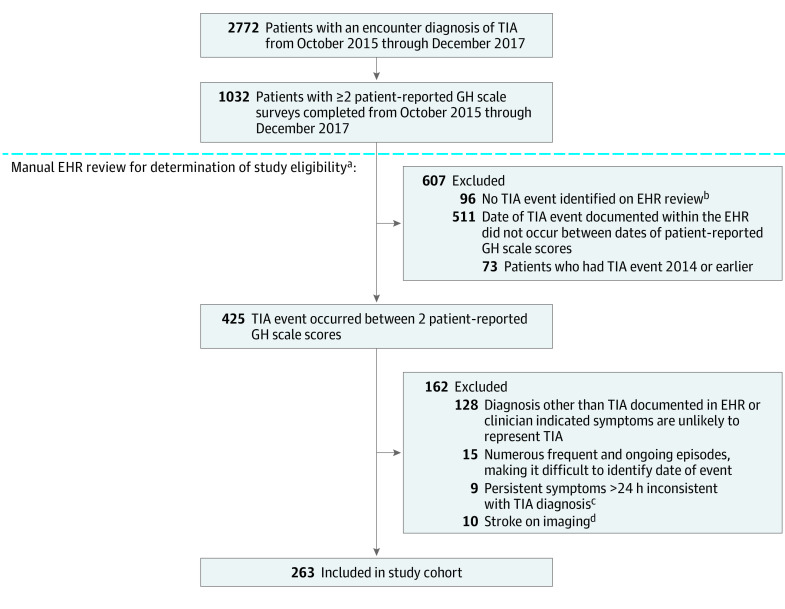
Patient Flowchart EHR indicates electronic health record; GH, global health; TIA, transient ischemic attack. ^a^This included review of the date that the event occurred (which may have been different than date of encounter in which TIA was recorded) and other inclusion and exclusion criteria. ^b^In these instances, TIAs were often entered as encounter diagnoses at the time that diagnostic tests were ordered. ^c^Persistent symptoms lasting days often consisted of dizziness, mental status change, or feeling weak. ^d^This was used in sensitivity analysis to evaluate the ability of GH scores to detect change.

**Table 1.** Demographic and Clinical Characteristics at Time of Index TIA

Characteristic	Missing values, No.	Patients, No. (%) (N = 263)
Age at initial TIA, mean (SD) [range], y	0	67.9 (13.4) [21-94]
Women	0	138 (52.5)
Men	0	125 (47.5)
Race		
White	7	223 (87.1)
Black		28 (10.9)
Other		5 (2.0)
Household income (per $10 000), median (IQR), $^a^	1	6.20 (4.97-7.22)
Marital status		
Married	5	160 (62.0)
Single		34 (13.2)
Divorced		21 (8.1)
Widowed		43 (16.7)
BMI, mean (SD)	0	29.4 (6.1)
Visit type for index TIA		
Outpatient	0	57 (21.7)
Emergency		82 (31.2)
Inpatient		120 (45.6)
Unknown or NA		4 (1.5)
Nonneurological clinical impression at index TIA		
TIA	0	NA
Probable		112 (42.6)
Possible		75 (28.5)
Unlikely		13 (4.9)
Unable to determine, as written by clinician		5 (1.9)
Unknown or unidentified		15 (5.7)
NA (ie, none recorded or no nonneurological clinician)		43 (16.3)
Neurological clinical impression		
TIA	0	NA
Probable		101 (38.4)
Possible		82 (31.2)
Unlikely		30 (11.4)
Unable to determine as written by neurologist or unknown		6 (2.3)
NA (ie, no neurological clinician)		44 (16.7)
Pattern of deficits at index TIA		
Focal	0	147 (55.9)
Nonfocal		52 (19.8)
Mixed		62 (23.6)
Unable to determine		2 (0.8)
Stroke risk		
Total score, mean (SD) [range]	0	3.45 (1.56) [0-7]
Age ≥60 y	0	200 (76.0)
BP elevation^b^	NA	126 (47.9)
Initial BP, mean (SD)		
Systolic	48	145.5 (25.9)
Diastolic	49	76.8 (14.5)
Clinical features of TIA		
None or unable to determine	0	94 (35.7)
Speech impairment without weakness		89 (33.8)
Unilateral weakness		80 (30.4)
Duration of TIA, min		
<10 or unable to determine	0	105 (39.9)
10-59		63 (24.0)
≥60		95 (36.1)
Diabetes	0	79 (30.0)
Time between GH completion and TIA event, median (IQR), d		
Pre-event GH score to TIA event	0	75 (34-168)
TIA event to postevent GH score	0	47 (16-107)

Abbreviations: BMI, body mass index (calculated as weight in kilograms divided by height in meters squared); BP, blood pressure; IQR, interquartile range; NA, not applicable; GH, global health; TIA, transient ischemic attack.

^a^ Median income is by zip code.

^b^ Elevated systolic BP was defined as 140 mm Hg or greater and elevated diastolic BP as 90 mm Hg or greater. Response options were elevated BP and none or unknown.

The mean (SD) baseline PH score was 43.4 (8.2), which was statistically significantly decreased compared with the general population mean (SD) of 50 (10) (*P* < .001), and the difference was clinically relevant. The mean (SD) baseline MH score was 47.7 (9.7), which was statistically significantly decreased compared with the general population mean (SD) of 50 (10) (*P* < .001), but the difference was not clinically relevant (Table 2). The change in mean (SD) PH and MH scores after the event was not statistically significant (PH: 44.1 [8.2], for a mean [SE] improvement of 0.65 [0.38] points; *P* = .09; MH: 47.4 [9.1], for a mean [SE] worsening of 0.25 [0.38] points; *P* = .51). The proportions of patients with worsening or improvement that were clinically relevant after the event were similar for PH and MH summary scores (Table 2). There was statistically significant worsening in mean (SD) scores among 2 of 10 individual patient-reported GH scale GH item responses after vs before the event (general health: 2.89 [0.89] vs 2.99 (0.92); mean [SE] change, −0.10 (0.05); *P* = .03); pain: 3.60 [2.75] vs 4.22 [2.79]; mean [SE] change, −0.60 [0.18]; *P* < .001) (Table 3).

**Table 2.** Global Health Score Before vs After TIA

Summary score	Mean (SD)		Change, mean (SE)	*P* value	Patients with clinically relevant change,^a^ No. (%), (N = 263	
	Before TIA	After TIA			Worsening	Improvement
Global PH	43.4 (8.2)	44.1 (8.2)	0.65 (0.38)	.09	48 (18.3)	56 (21.3)
Global MH	47.7 (9.7)	47.4 (9.1)	−0.25 (0.38)	.51	54 (20.5)	41 (15.6)

Abbreviations: MH, mental health; PH, physical health; TIA, transient ischemic attack.

^a^ Clinically relevant change was defined as a change of 5 points or more.

**Table 3.** Global Health Scale Item Responses Before vs After TIA Among 263 Patients

Item^a^	Mean (SD)		Change, mean (SE)	*P* value
	Before TIA	After TIA		
1. General health	2.99 (0.92)	2.89 (0.89)	−0.10 (0.05)	.03
2. Quality of life^b^	3.23 (1.01)	3.26 (0.99)	0.03 (0.05)	.46
3. PH^c^	2.89 (0.94)	2.78 (0.92)	−0.09 (0.05)	.06
4. MH^b^	3.40 (1.09)	3.35 (1.07)	−0.04 (0.05)	.48
5. Social discretionary^b^	3.33 (1.08)	3.26 (1.12)	−0.07 (0.05)	.22
6. Physical function^c^	3.70 (1.13)	3.75 (1.14)	0.05 (0.06)	.38
7. Pain^c^	4.22 (2.79)	3.60 (2.75)	−0.60 (0.18)	<.001
8. Fatigue^c^	3.38 (0.90)	3.41 (0.84)	0.03 (0.05)	.48
9. Social roles	3.14 (1.10)	3.14 (1.14)	0.01 (0.05)	.89
10. Emotional problems^b^	3.62 (1.04)	3.58 (0.95)	−0.03 (0.05)	.52

Abbreviations: MH, mental health; PH, physical health; TIA, transient ischemic attack.

^a^ All items are on a scale from 1 to 5, with higher scores indicating better health.

^b^ This question is part of the MH summary score.

^c^ This question is part of the PH summary score.

There was no statistically significant difference in pre-event patient-reported GH scale summary scores between categories in the following prespecified subgroups: clinicians’ impression of probability of TIA, pattern of deficits, and short-term risk of stroke (ie, stroke risk score ≥4) (Table 4). Additionally, there was no statistically significant difference in the proportion of patients who experienced a change of 5 or greater in patient-reported GH scale summary scores for categories in these subgroups.

**Table 4.** GH Summary Score by Subgroup

GH score, mean (SD)	Neurological clinical impression			Nonneurological impression			Pattern of neurological deficit				Risk of future stroke^a^		
	TIA		*P* value	TIA		*P* value	Focal (n = 147)	Nonfocal (n = 52)	Mixed (n = 62)	*P* value	Low (n = 128)	Moderate or high (n = 135)	*P* value
	Possible (n = 82)	Probable (n = 101)		Possible (n = 75)	Probable (n = 112)								
**Before event**													
PH	43.6 (8.1)	42.6 (8.6)	.43	43.2 (7.7)	43.0 (8.5)	.87	43.2 (7.8)	44.6 (8.5)	43.2 (8.9)	.54	44.1 (8.7)	42.7 (7.7)	.17
MH	46.7 (9.6)	48.4 (9.2)	.25	47.5 (9.3)	47.5 (10.3)	.99	48.1 (9.3)	48.5 (11.3)	46.2 (9.0)	.36	47.9 (10.2)	47.4 (9.1)	.71
**After event, mean (SD)**													
PH	43.7 (8.4)	43.9 (8.7)	.84	43.7 (7.7)	43.4 (8.4)	.83	43.7 (8.0)	45.1 (7.5)	43.7 (8.7)	.51	45.1 (8.4)	43.1 (7.9)	.06
MH	45.7 (9.3)	48.6 (8.7)	.03	46.4 (8.1)	47.3 (9.8)	.52	47.9 (9.1)	48.5 (9.5)	45.0 (8.1)	.07	47.9 (9.6)	47.0 (8.5)	.43
**Change in GH score**^b^													
PH													
Change in score, mean (SE)^c^	0.10 (0.61)	1.35 (0.59)^d^	.15	0.49 (0.68)	0.43 (0.65)	.95	0.50 (0.50)	0.53 (0.93)	0.51 (0.62)	.999	0.92 (0.59)	0.40 (0.50)	.50
Improvement, No. (%)	12 (14.6)	26 (25.7)	.07	13 (17.3)	24 (21.4)	.49	29 (19.7)	13 (25.0)	12 (19.4)	.69	32 (25.0)	24 (17.8)	.15
Worsening, No. (%)	15 (18.3)	17 (16.8)	.80	11 (14.7)	25 (22.3)	.19	29 (19.7)	11 (21.2)	8 (12.9)	.43	19 (14.8)	29 (21.5)	.16
MH													
Change in score, mean (SE)^c^	−1.01 (0.62)	0.29 (0.54)	.11	−1.13 (0.73)	−0.24 (0.62)	.36	−0.18 (0.44)	0.04 (1.06)	−1.18 (0.69)	.45	−0.03 (0.52)	−0.46 (0.55)	.57
Improvement, No. (%)	9 (11.0)	17 (16.8)	.26	9 (12.0)	19 (17.0)	.35	23 (15.7)	12 (23.1)	5 (8.1)	.09	21 (16.4)	20 (14.8)	.72
Worsening, No. (%)	20 (24.4)	17 (16.8)	.21	23 (30.7)	22 (19.6)	.08	28 (19.1)	14 (26.9)	12 (19.4)	.46	23 (18.0)	31 (23.0)	.32

Abbreviations: GH, global health; MH, mental health; PH, physical health; TIA, transient ischemic attack.

^a^ Stroke risk score (ie, ABCD2 score) was defined as low risk at less than 4 and moderate or high risk at 4 or greater.

^b^ Clinically relevant change was defined as a change of 5 points or more.

^c^ Positive change indicates improvement; negative change indicates worsening.

^d^ Statistically significant change from before to after events was based on paired *t* test.

There was a statistically significant improvement in PH scores from before the event to after the event among patients with at least 1 medication change of any type, although this difference was not clinically relevant (mean [SD], 42.9 (8.2) vs 43.8 (8.3); mean [SE] change, 0.88 [0.42] points; *P* = .03) (eTable 2 in the Supplement). Although numbers within each category were small (eg, 65 patients with changes to pain medications, 131 patients with changes to antithrombotic medications, and 36 patients with changes to antimicrobial medications), there were no statistically significant changes in patient-reported GH scale summary scores with medication changes in the prespecified medication categories.

In univariate analysis, independent variables associated with clinically relevant worsening in PH scores included increased stroke risk score (odds ratio [OR] per each 1-unit increase in score, 1.26; 95% CI, 1.02-1.55; *P* = .03), hypertension among 188 patients (OR, 3.31; 95% CI, 1.34-8.15; *P* = .009), and interval hospital admission among 35 patients (OR, 2.37; 95% CI, 1.07-5.24; *P* = .03) (eTable 3 in the Supplement). Independent variables associated with worsening patient-reported GH scale MH scores included outpatient evaluation of TIA among 57 patients (OR, 2.31; 95% CI, 1.10-4.82; *P* = .03), interval stroke among 4 patients (OR, 12.2; 95% CI, 1.25-120.1; *P* = .03), and interval hospital admission (OR, 2.31; 95% CI, 1.07-5.01; *P* = .03).

In multivariable logistic regressions, stroke risk score (OR per each 1-unit increase in score, 1.34; 95% CI, 1.05–1.71; *P* = .02) and male sex (OR, 2.18; 95% CI, 1.05-4.54; *P* = .04) were independently associated with clinically relevant worsening of PH score, while being married was associated with lower odds of worsening PH score (OR, 0.49; 95% CI, 0.24-0.98; *P* = .04) (eTable 4 in the Supplement). The presence of nonfocal symptoms was associated with clinically relevant worsening in patient-reported GH scale MH score (OR, 2.39; 95% CI, 1.02-5.63; *P* = .046).

In the sensitivity analysis to assess for selection bias, we compared the mean postevent patient-reported GH scale scores among patients seen in the cerebrovascular clinic who had pre-event patient-reported GH scale scores with the postevent GH scores among patients who did not have pre-event GH scores (and so were not included in the study cohort). Among patients with pre-event scores, compared with those without pre-event scores, the mean (SD) postevent patient-reported GH scale scores were 44.5 (9.1) vs 45.1 (9.0) for PH, for a difference of 0.6 points, and 47.8 (8.8) vs 48.2 (9.1) for MH, for a difference of 0.4 points; these differences were not clinically relevant (eTable 5 in the Supplement). In the sensitivity analysis to determine the ability of patient-reported GH scale to detect change, PH and MH scores of 10 patients with transient symptoms excluded from the study owing to the presence of an acute infarct on MRI decreased to a greater degree compared with study patients (eTable 6 in the Supplement). The third sensitivity analysis assessed patient-reported GH scale scores among 158 patients with MRIs as part of their initial TIA evaluation. Results were similar to those of the full cohort (eTable 7 in the Supplement).

## Discussion

In this cohort study of patient-reported health before and after TIA, patients with a clinical diagnosis of TIA had baseline patient-reported PH that was worse by a clinically relevant degree compared with the US population. Patient-reported MH and PH did not change significantly after the event. Our findings suggest that preexisting health status is at least partially associated with the impaired health status found among patients diagnosed with TIA.

Not unexpectedly, given the poor PH scores prior to the TIA event, patients had high rates of risk factors associated with vascular events and other comorbid conditions. This finding is intuitive, given that vascular risk factors associated with TIA may also be associated with cognitive impairment^3^ and depression.^25^ There may be another contributing factor. In prior work,^26^ our group found that, after adjustment for disability and other factors, patients with a TIA diagnosed by vascular neurologists had significantly worse self-reported health compared with patients with ischemic stroke, subarachnoid hemorrhage, or intracerebral hemorrhage. This raises the possibility that some patients diagnosed with TIA have a noncerebrovascular etiology of symptoms. Indeed, agreement in the clinical diagnosis of TIA among neurologists is modest at best.^27,28^ Because of this, the term transient neurological attack has been coined to describe episodes of transient neurological symptoms that do not suggest an underlying mechanism.^20^ Patients with transient neurological symptoms that are nonischemic in origin may have other conditions that negatively impact their health.^3^

In the multivariable model, we found that patients with nonfocal symptoms were more likely to have clinically relevant worsening (ie, decreases of 5 points or more) in patient-reported GH scale MH score, while greater stroke risk scores (which suggest an ischemic etiology) were associated with clinically meaningful worsening in patient-reported GH scale PH score. Although this may suggest that nonvascular etiologies of transient symptoms are associated with worsening of MH and ischemic events are associated with worsening of PH, these results should be interpreted cautiously. Recent evidence suggests that transient symptoms traditionally considered to suggest a diagnosis other than stroke are not associated with a decreased risk of acute infarct on DWI-MRI^29,30^ and limitations to the predictive value of stroke risk score have been noted in the last several years.^31^ Perhaps the most appropriate interpretation of available data, then, is that the ability to make an accurate diagnosis of TIA based on clinical event characteristics is poor. This may explain why patients with episodes that are classified as transient neurological attacks are also at increased risk of cardiovascular morbidity and mortality.^20,32^ Given the difficulty with accurate clinical diagnosis of TIA, it seems prudent to consider MRI and strive for careful control of risk factors associated with stroke among these patients.

Several other factors potentially associated with impaired health status seen among patients with TIA diagnosis were explored in this study. There was no association between medication changes and worsening of patient-reported GH scale scores. In univariate analysis, there were higher odds of worsening of PH and MH scores among 35 patients who had an interval hospital admission and higher odds of meaningful worsening in MH scores among 4 patients who had interval strokes. Our findings are consistent with prior studies^4,8,9^ that found subsequent stroke to be associated with poor HRQOL after TIA, although our study suggests that nonstroke medical events leading to hospital admission are more commonly associated with poor HRQOL.

An important consideration when interpreting this study is the responsiveness of the patient-reported GH scale to detect changes. Previous evaluation has demonstrated moderate to large effect sizes for change in the patient-reported GH scale score among patients with stroke.^33^ The sensitivity analysis performed to evaluate the ability of the patient-reported GH scale to detect change in our study cohort supports the responsiveness of this measure; the mean declines in PH and MH scores among patients with transient symptoms excluded from the study owing to stroke on imaging were greater than the minimal score changes seen in our study cohort.

This study provides new and important insights about the outcomes among patients with clinical diagnoses of TIA. A unique strength of our study is the availability of baseline HRQOL, allowing a direct assessment of change over time. Detailed EHR review was performed to include only events with a clinical impression of TIA.

### Limitations

This study also has several limitations. First, there was likely a selection bias among patients with TIA who had a pre-event patient-reported GH scale score. Reassuringly, our sensitivity analysis suggested that patients seen in the cerebrovascular clinic without pre-event patient-reported GH scale scores had postevent patient-reported GH scale scores that were similar to those of patients who had pre-event scores within that clinic. Second, this study consisted of modest patient numbers from 1 institution, limiting our ability to detect change, particularly within subgroups. Third, the patient-reported GH scale may be insensitive to subtle changes or constructs not covered within the scale. Further study is needed on additional outcome measures. Fourth, 60% of patients had MRI at the time of the event, and it is possible that some patients in the cohort had an unidentified stroke, possibly confounding our results. Importantly, however, sensitivity analysis of patients with MRI at the time of the event had similar findings compared with the full cohort.

## Conclusions

These findings suggest that the impaired HRQOL found among patients diagnosed with TIA without imaging evidence of structural damage reflect, at least in part, an impaired premorbid state of health. We found that TIA events were not associated with worsening of health status overall, although they were associated with worsening on 2 of 10 GH scale items. However, interval stroke and hospital admission were associated with clinically relevant worsening. Further evaluation is necessary to confirm these findings using different patient-reported outcomes and objective performance measures.

## Notes

Supplement. **eTable 1.** Comorbidities Prior to Baseline Global Health and Between Baseline and Post-Event Global Health (N = 263) **eTable 2.** Global Health Scores Among Patients With Medication Changes **eTable 3.** Univariate Factors Associated With Clinically Relevant Improvement and Worsening in Global Physical and Mental Health **eTable 4.** Multivariable Logistic Regression Analysis of Clinically Relevant Worsening in Global Health Summary Scores After Transient Ischemic Attack **eTable 5.** Global Health Scores After Transient Ischemic Attack Among Patients Seen in Cerebrovascular Center **eTable 6.** Global Health Scores Among 10 Patients Excluded Owing to Stroke on Magnetic Resonance Imaging **eTable 7.** Global Health Scores Among 158 Patients With Magnetic Resonance Imaging in Transient Ischemic Attack Evaluation
