# Supplementary material for: Changes in Health-Related Quality of Life After Transient Ischemic Attack
Source: JAMA Netw Open. 2021 Jul 20;4(7):e2117403. doi: 10.1001/jamanetworkopen.2021.17403 (PMC8293018; doi:10.1001/jamanetworkopen.2021.17403)
Supplement: Supplement. — eTable 1. Comorbidities Prior to Baseline Global Health and Between Baseline and Post-Event Global Health (N = 263) eTable 2. Global Health Scores Among Patients With Medication Changes eTable 3. Univariate Factors Associated With Clinically Relevant Improvement and Worsening in Global Physical and Mental Health eTable 4. Multivariable Logistic Regression Analysis of Clinically Relevant Worsening in Global Health Summary Scores After Transient Ischemic Attack eTable 5. Global Health Scores After Transient Ischemic Attack Among Patients Seen in Cerebrovascular Center eTable 6. Global Health Scores Among 10 Patients Excluded Owing to Stroke on Magnetic Resonance Imaging eTable 7. Global Health Scores Among 158 Patients With Magnetic Resonance Imaging in Transient Ischemic Attack Evaluation [file jamanetwopen-e2117403-s001.pdf]

Supplemental Online Content

Katzan IL, Schuster A, Daboul L, et al. Changes in health-related quality of life after transient ischemic attack. *JAMA Netw Open*. 2021;4(7):e2117403. doi:10.1001/jamanetworkopen.2021.17403

eTable 1. Comorbidities Prior to Baseline Global Health and Between Baseline and Post-Event Global Health (N = 263)
eTable 2. Global Health Scores Among Patients With Medication Changes
eTable 3. Univariate Factors Associated With Clinically Relevant Improvement and Worsening in Global Physical and Mental Health
eTable 4. Multivariable Logistic Regression Analysis of Clinically Relevant Worsening in Global Health Summary Scores After Transient Ischemic Attack
eTable 5. Global Health Scores After Transient Ischemic Attack Among Patients Seen in Cerebrovascular Center
eTable 6. Global Health Scores Among 10 Patients Excluded Owing to Stroke on Magnetic Resonance Imaging
eTable 7. Global Health Scores Among 158 Patients With Magnetic Resonance Imaging in Transient Ischemic Attack Evaluation

This supplemental material has been provided by the authors to give readers additional information about their work.

© 2021 Katzan IL et al. *JAMA Network Open.*

eTable 1. Comorbidities Prior to Baseline Global Health and Between Baseline and Post-Event Global Health (N = 263)

Comorbidities	Prior to Baseline PROMIS GH, N (%)	Between Pre- and Post-event PROMIS GH, N (%)
TIA	74 (28.1)	15 (5.7)
Ischemic or hemorrhagic stroke	52 (19.8)	4 (1.5)
Previous stroke/TIA treatments or procedures	13 (4.9)	n/a
Mechanical thrombectomy	0	
Endarterectomy	8 (3.0)	
Aneurysm embolization (clip/coil)	1 (0.4)	
Arteriovenous malformation treatment	1 (0.4)	
Bypass	1 (0.4)	
Hemicraniectomy or cranioplasty	2 (0.8)	
Previous visit to Cerebrovascular Center	33 (12.5)	n/a
Smoking		-
Never	126 (47.9)	
Past	112 (42.6)	
Current	25 (9.5)	
Hypertension	188 (71.5)	-
Depression	79 (30.0)	15 (5.7)
Anxiety	52 (19.8)	2 (0.8)
Mental health or cognitive disorder (other than depression/anxiety)[a]	25 (9.5)	-
Atrial fibrillation	41 (15.6)	-
Chronic kidney disease	38 (14.4)	2 (0.8)
Coronary artery disease	64 (24.3)	-
Heart failure	23 (8.7)	1 (0.4)
Chronic obstructive pulmonary disease	24 (9.1)	2 (0.8)
Asthma	35 (13.3)	1 (0.4)
Cancer	67 (25.5)	6 (2.3)
Trauma	-	6 (2.3)
Chronic pain or neurological disorder	217 (82.5)	78 (29.7)
Low back pain/sciatica/spinal stenosis	115 (43.7)	22 (8.4)
Arthritis (rheumatoid/osteoarthritis)	94 (35.7)	13 (4.9)
Headache/migraine	50 (19.0)	22 (8.4)
Multiple sclerosis	2 (0.8)	1 (0.4)
Myasthenia gravis	0	0
Vasculitis	1 (0.4)	1 (0.4)
Fibromyalgia	11 (4.2)	1 (0.4)
Neuropathy	27 (10.3)	6 (2.3)
Other	82 (31.2)	36 (13.7)

PROMIS GH = PROMIS Global Health; TIA = transient ischemic attack; a--Other mental health or cognitive disorders includes bipolar disorder (n=10), dementia (n=7), schizophrenia (n=2), and other (n=8)

© 2021 Katzan IL et al. *JAMA Network Open.*

eTable 2. Global Health Scores Among Patients With Medication Changes

Score	Pre-TIA Mean (SD)	Post-TIA Mean (SD)	Change Mean (SE)	P-Value
**No Medication Changes, n=26**				
PROMIS Global Physical Health	47.9 (7.0)	46.4 (6.9)	-1.47 (0.80)	0.079
PROMIS Global Mental Health	51.3 (7.6)	49.9 (8.0)	-1.47 (0.94)	0.132
ANY Medication Changes, n=237				
PROMIS Global Physical Health	**42.9 (8.2)**	**43.8 (8.3)**	**0.88 (0.42)**	**0.034**
PROMIS Global Mental Health	47.3 (9.8)	47.1 (9.2)	-0.12 (0.41)	0.776
**CNS drug changes, n=84**				
PROMIS Global Physical Health	40.0 (7.1)	41.2 (7.8)	1.20 (0.71)	0.097
PROMIS Global Mental Health	43.5 (9.0)	43.4 (7.9)	-0.07 (0.66)	0.917
**GI drug changes, n=38**				
PROMIS Global Physical Health	39.3 (7.4)	39.9 (7.3)	0.64 (0.99)	0.523
PROMIS Global Mental Health	42.5 (8.4)	43.1 (6.3)	0.63 (0.79)	0.430
**OTC drug changes, n=49**				
PROMIS Global Physical Health	41.3 (7.3)	41.4 (7.5)	0.13 (0.83)	0.880
PROMIS Global Mental Health	44.8 (8.1)	46.0 (8.2)	1.18 (0.86)	0.178
**Antimicrobial drug changes, n=36**				
PROMIS Global Physical Health	42.9 (7.4)	43.9 (7.0)	1.01 (1.16)	0.390
PROMIS Global Mental Health	46.9 (8.0)	46.3 (5.8)	-0.56 (1.30)	0.670
**Antithrombotic drug changes, n=131**				
PROMIS Global Physical Health	43.7 (8.6)	44.5 (9.0)	0.75 (0.62)	0.227
PROMIS Global Mental Health	48.9 (10.5)	48.7 (10.0)	-0.18 (0.59)	0.757
**Cardiac drug changes, n=123**				
PROMIS Global Physical Health	42.3 (8.2)	43.3 (7.9)	1.01 (0.62)	0.109
PROMIS Global Mental Health	47.4 (9.6)	48.1 (9.2)	0.74 (0.58)	0.205
**Diabetic medication changes, n=9**				
PROMIS Global Physical Health	41.2 (9.4)	41.1 (8.2)	-0.07 (1.94)	0.973
PROMIS Global Mental Health	43.7 (12.6)	44.5 (10.3)	0.88 (2.10)	0.687
**Hormone drug changes, n=14**				
PROMIS Global Physical Health	42.8 (6.4)	43.4 (6.4)	0.59 (1.45)	0.693
PROMIS Global Mental Health	46.6 (7.8)	45.6 (6.7)	-1.03 (1.68)	0.551
**Immunomodulating drug changes, n=23**				
PROMIS Global Physical Health	40.0 (7.5)	40.2 (8.6)	0.24 (1.65)	0.886
PROMIS Global Mental Health	46.2 (9.6)	46.7 (10.7)	0.52 (1.23)	0.677
**Other drug changes, n=29**				
PROMIS Global Physical Health	42.1 (7.6)	41.7 (7.2)	-0.34 (1.11)	0.761
PROMIS Global Mental Health	46.5 (9.3)	46.0 (10.0)	-0.44 (1.13)	0.700
**Pain med changes, n=65**				
PROMIS Global Physical Health	41.9 (7.2)	41.6 (6.3)	-0.27 (0.78)	0.734
PROMIS Global Mental Health	47.3 (8.9)	46.8 (8.7)	-0.56 (0.85)	0.512
**Respiratory med changes, n=22**				

© 2021 Katzan IL et al. *JAMA Network Open.*

PROMIS Global Physical Health	41.2 (7.7)	43.2 (8.9)	2.00 (1.62)	0.232
PROMIS Global Mental Health	44.0 (10.6)	47.1 (10.5)	3.06 (1.60)	0.069
**Sedative/hypnotic - CNS drug changes, n=23**				
PROMIS Global Physical Health	46.5 (6.7)	46.5 (7.5)	-0.004 (1.41)	0.998
PROMIS Global Mental Health	47.7 (9.6)	46.3 (8.1)	-1.42 (1.57)	0.375
**Topical drug changes, n=4**				
PROMIS Global Physical Health	36.1 (1.4)	40.5 (5.5)	4.32 (2.35)	0.163
PROMIS Global Mental Health	46.1 (7.8)	38.1 (2.3)	-7.92 (3.24)	0.092

© 2021 Katzan IL et al. *JAMA Network Open.*

eTable 3. Univariate Factors Associated With Clinically Relevant Improvement and Worsening in Global Physical and Mental Health

	Worsening GPH (n=48)		Worsening GMH (n=54)	
	**Odds Ratio (95% CI)**	**P-Value**	**Odds Ratio (95% CI)**	**P-Value**
**Demographics and Clinical Characteristics at Time of Index TIA**				
Age (per decade)	1.14 (0.89-1.46)	0.299	1.18 (0.93-1.50)	0.172
Male (vs female)	**1.89 (1.00-3.58)**	**0.050**	1.24 (0.68-2.26)	0.476
Non-white race (vs white)	1.20 (0.49-2.94)	0.698	1.34 (0.57-3.18)	0.507
Household income (per $10k)	1.01 (0.86-1.19)	0.912	1.05 (0.90-1.23)	0.524
Married (vs non-married)	0.61 (0.32-1.16)	0.131	1.49 (0.78-2.85)	0.232
BMI (per kg/m2)	1.01 (0.96-1.06)	0.806	0.99 (0.94-1.04)	0.600
Visit type				
Outpatient (vs Inpatient)	0.96 (0.43-2.12)	0.913	**2.31 (1.10-4.82)**	**0.026**
Emergency (vs Inpatient)	0.75 (0.36-1.58)	0.455	1.12 (0.54-2.34)	0.764
Non-neurology impression, probable TIA (vs non-probable)	1.67 (0.77-3.64)	0.196	0.55 (0.28-1.09)	0.086
Neurology clinical impression, probable TIA (vs non-probable)	0.90 (0.42-1.94)	0.796	0.63 (0.30-1.30)	0.208
Pattern of deficits (at index TIA)				
Focal (vs Mixed)	1.66 (0.71-3.87)	0.241	0.98 (0.46-2.08)	0.959
Nonfocal (vs Mixed)	1.81 (0.67-4.91)	0.243	1.54 (0.64-3.70)	0.339
$ABCD^2$ total score	**1.26 (1.02-1.55)**	**0.030**	1.13 (0.93-1.38)	0.213
Moderate/high risk of future stroke ($ABCD^2$ 4+ vs $ABCD^2$ <4)	1.57 (0.83-2.97)	0.166	1.36 (0.74-2.49)	0.317
<u>$ABCD^2$ components:</u>				
Age ≥60 years	1.45 (0.66-3.19)	0.352	2.05 (0.91-4.62)	0.082
BP elevation (systolic ≥140 or diastolic ≥90 mmHg)	1.36 (0.72-2.55)	0.338	1.11 (0.61-2.02)	0.730
Clinical features of TIA (reference = None/unable to determine)				
Speech impairment without weakness	1.16 (0.52-2.56)	0.717	1.24 (0.59-2.61)	0.578
Unilateral weakness	1.78 (0.83-3.83)	0.140	1.63 (0.78-3.40)	0.198
Duration of TIA (reference <10 minutes/unable to determine)				
10-59 minutes	2.21 (0.97-5.02)	0.058	0.93 (0.43-1.99)	0.846
≥ 60 minutes	1.89 (0.88-4.04)	0.102	0.83 (0.42-1.66)	0.605
Diabetes, n (%)	0.95 (0.48-1.89)	0.884	1.35 (0.72-2.54)	0.356
**Comorbidities Prior to Pre-TIA PROMIS GH**				
TIA	1.35 (0.69-2.65)	0.377	1.69 (0.90-3.18)	0.105
Ischemic or hemorrhagic stroke	0.64 (0.27-1.53)	0.321	0.65 (0.29-1.48)	0.308
Previous stroke/TIA treatments or procedures	0.81 (0.17-3.76)	0.784	0.69 (0.15-3.22)	0.640
Previous visit to CV center	0.58 (0.19-1.74)	0.335	1.05 (0.43-2.56)	0.918
Smoking				
Past (vs never)	1.62 (0.84-3.13)	0.153	1.29 (0.69-2.40)	0.432

© 2021 Katzan IL et al. *JAMA Network Open.*

Current (vs never)	1.07 (0.33-3.47)	0.907	0.81 (0.25-2.58)	0.721
Hypertension	**3.31 (1.34-8.15)**	**0.009**	0.93 (0.48-1.80)	0.839
Depression	0.74 (0.36-1.50)	0.401	0.69 (0.34-1.37)	0.286
Anxiety	0.64 (0.27-1.53)	0.321	1.05 (0.50-2.21)	0.901
Mental health or cognitive disorder (not depression/anxiety)	1.13 (0.40-3.19)	0.812	0.50 (0.14-1.74)	0.275
Atrial fibrillation	0.91 (0.38-2.19)	0.832	0.62 (0.25-1.56)	0.312
Chronic kidney disease	1.48 (0.65-3.37)	0.350	1.04 (0.45-2.42)	0.931
Coronary artery disease	0.67 (0.31-1.47)	0.321	1.11 (0.56-2.21)	0.760
Heart failure	1.27 (0.45-3.62)	0.651	0.80 (0.26-2.46)	0.697
Chronic obstructive pulmonary disease	1.99 (0.77-5.10)	0.153	1.02 (0.36-2.87)	0.969
Asthma	0.92 (0.36-2.35)	0.855	0.61 (0.22-1.65)	0.330
Cancer	1.81 (0.92-3.54)	0.083	1.30 (0.67-2.53)	0.433
Chronic pain or neurological disorder	1.30 (0.54-3.11)	0.559	1.08 (0.48-2.39)	0.858
ED visit	1.36 (0.55-3.37)	0.508	0.92 (0.36-2.37)	0.863
Hospital admissions	0.78 (0.28-2.13)	0.623	1.54 (0.67-3.55)	0.308
**Comorbidities between Pre-to Post-TIA PROMIS GH**				
TIA	0.31 (0.04-2.38)	0.258	2.03 (0.66-6.21)	0.214
Ischemic or hemorrhagic stroke	1.50 (0.15-14.8)	0.726	**12.2 (1.25-120.1)**	**0.032**
Depression	1.13 (0.31-4.16)	0.857	1.44 (0.44-4.71)	0.546
Chronic pain or neurological disorder	0.97 (0.49-1.93)	0.935	0.99 (0.52-1.92)	0.996
ED visit	1.36 (0.55-3.37)	0.508	1.15 (0.47-2.83)	0.764
Hospital admissions	**2.37 (1.07-5.24)**	**0.034**	**2.31 (1.07-5.01)**	**0.034**
**Months between PROMIS GH Scores**				
Between Pre-TIA PROMIS GH and TIA	0.99 (0.91-1.07)	0.787	1.05 (0.98-1.13)	0.155
Between TIA and Post-TIA PROMIS GH	0.96 (0.87-1.06)	0.416	1.00 (0.92-1.09)	0.953
Between scores	0.98 (0.92-1.04)	0.454	1.03 (0.98-1.09)	0.266

Meaningful change was defined as 5+ change in PROMIS GH summary score post-TIA event. Refer to Supplemental Table 2 for number of patients experiencing change in medical conditions between PROMIS GH completions

© 2021 Katzan IL et al. *JAMA Network Open.*

eTable 4. Multivariable Logistic Regression Analysis of Clinically Relevant Worsening in Global Health Summary Scores After Transient Ischemic Attack

**Predictors**	**PROMIS Global Physical Health**		**PROMIS Global Mental Health**	
	**Odds Ratio (95% CI)**	**P-Value**	**Odds Ratio (95% CI)**	**P-Value**
Age (per decade)	0.97 (0.73-1.27)	0.806	1.07 (0.80-1.42)	0.648
Male (vs female)	**2.18 (1.05-4.54)**	**0.036**	1.59 (0.79-3.19)	0.192
Non-white race (vs white)	1.14 (0.42-3.11)	0.799	1.59 (0.61-4.13)	0.344
Married (vs non-married)	**0.49 (0.24-0.98)**	**0.044**	1.76 (0.86-3.61)	0.125
Pattern of deficits (at index TIA)				
Mixed (vs Focal)	0.70 (0.29-1.72)	0.438	1.21 (0.53-2.75)	0.644
Nonfocal (vs Focal)	1.87 (0.76-4.58)	0.169	**2.39 (1.02-5.63)**	**0.046**
$ABCD^2$ total score	**1.34 (1.05-1.71)**	**0.017**	1.12 (0.89-1.41)	0.348
Months between PROMIS GH scores	0.98 (0.91-1.05)	0.510	1.04 (0.98-1.10)	0.180

© 2021 Katzan IL et al. *JAMA Network Open.*

Sensitivity Analyses

eTable 5. Global Health Scores After Transient Ischemic Attack Among Patients Seen in Cerebrovascular Center

Score	Post-TIA with a Pre-TIA Score Mean (SD)	Post-TIA[a] without a Pre-TIA score Mean (SD)	All Post-TIA Mean (SD)
PROMIS Global Physical Health	44.5 (9.1) [n=108]	45.1 (9.0) [n=554]	45.0 (9.0) [n=662]
PROMIS Global Mental Health	47.8 (8.8) [n=114]	48.2 (9.1) [n=573]	48.1 (9.1) [n=687]

SD = standard deviation. a-patients without pre-TIA score were excluded from the study cohort but were used in sensitivity analysis to evaluate representativeness of study population for all patients with an encounter diagnosis of TIA

© 2021 Katzan IL et al. *JAMA Network Open.*

eTable 6. Global Health Scores Among 10 Patients Excluded Owing to Stroke on Magnetic Resonance Imaging

Score	Pre-TIA Mean (SD)	Post-TIA Mean (SD)	Change Mean (SE)	P-Value
PROMIS Global Physical Health	42.0 (6.9)	37.8 (6.3)	-4.3 (2.1)	0.075
PROMIS Global Mental Health	46.8 (10.2)	40.9 (7.4)	-5.9 (3.7)	0.140

SD = standard deviation; SE = standard error

These 10 patients had an encounter diagnosis of TIA but were excluded from the study due to the presence of an acute infarct on MRI. They were included in a sensitivity analysis to evaluate responsiveness of PROMIS GH to detect change in patients with transient symptoms but with stroke on imaging

© 2021 Katzan IL et al. *JAMA Network Open.*

eTable 7. Global Health Scores Among 158 Patients With Magnetic Resonance Imaging in Transient Ischemic Attack Evaluation

Score	Pre-TIA Mean (SD)	Post-TIA Mean (SD)	Change Mean (SE)	P-Value
PROMIS Global Physical Health	42.7 (8.5)	43.7 (8.3)	0.98 (0.51)	0.055
PROMIS Global Mental Health	47.8 (9.8)	48.0 (9.2)	0.17 (3.51)	0.744

SD = standard deviation; SE = standard error

The subgroup of patients with DWI MRI done for evaluation of TIA were included in a sensitivity analysis to evaluate for possible confounding by including patients in the study cohort without MRI at the time of the event who therefore may have had an undiagnosed stroke on imaging

© 2021 Katzan IL et al. *JAMA Network Open.*
